# The use of volatile anesthetic agents for long-term critical care sedation (VALTS): study protocol for a pilot randomized controlled trial

**DOI:** 10.1186/s13063-015-1083-5

**Published:** 2015-12-09

**Authors:** Angela Jerath, Niall D. Ferguson, Andrew Steel, Duminda Wijeysundera, John Macdonald, Marcin Wasowicz

**Affiliations:** Department Anesthesia and Pain Medicine, Toronto General Hospital, 200 Elizabeth St, Toronto, Ontario M5G 2C4 Canada; Department Critical Care Medicine, Toronto General Hospital, 200 Elizabeth St, Toronto, Ontario M5G 2C4 Canada; Department Critical Care Medicine and Anesthesia, Toronto General Hospital, 200 Elizabeth St, Toronto, Ontario M5G 2C4 Canada; Department Anesthesia, University of Ottawa Heart Institute, 40 Ruskin St, Ottawa, Ontario K1Y 4W7 Canada

**Keywords:** Volatile anesthetics, sedation, critical care, safety, randomized controlled trial

## Abstract

**Background:**

Sedatives are administered to 85 % of intensive care unit (ICU) patients. The most commonly used sedatives are intravenous benzodiazepines and propofol. These agents are associated with over-sedation in 40 to 60 % of patients, which can lead to prolonged intubation, delirium and drug-induced hypotension. Evidence is increasing that volatile anesthetic agents are associated with faster extubation times, improved cardiovascular stability with no end-organ toxicity in comparison to our standard intravenous agents for short-term critical care sedation. Use of volatile agents within the ICU is a novel technique using a specialized delivery and scavenging system, which requires staff training and cultural acceptance. This pilot randomized controlled trial aims to assess the safety and feasibility of delivering volatile agents for long-term patient sedation in the ICU with limited or no experience of this technique.

**Methods/Design:**

This is a prospective multicenter pragmatic pilot RCT that is blinded to the data analyst. This study aims to recruit 60 adult ICU patients requiring mechanical ventilation and sedation for more than 48 h. Patients will be randomized 2:1 to receive inhaled isoflurane (40 patients) or intravenous midazolam and/or propofol (20 patients) sedation. Sedation is titrated to a targeted Sedation Analgesia Score (SAS) using an explicit sedation-analgesia algorithm until extubation or tracheostomy. Primary safety and feasibility outcomes will assess atmospheric volatile concentration levels and adherence to our sedation-analgesia protocol. Secondary outcomes include time to extubation, duration of ventilation, quality of sedation, delirium, vasoactive drug support, length of stay, serum fluoride levels and mortality.

**Discussion:**

This pilot project will serve as the basis for a larger RCT that will be powered for important clinical outcomes.

**Trial Registration:**

ClinicalTrials.gov, NCT01983800 (registration date 2 July 2013).

## Background

Sedation is a cornerstone of patient care within intensive care units (ICU). Sedative and analgesic medications are administered to 85 % of ICU patients to assist tolerance of mechanical ventilation and invasive procedures and to treat anxiety and pain [1]. Current national and international sedation practice predominantly uses systemic opioids to provide analgesia combined with intravenous benzodiazepines (BDZ) in 60 % and propofol in 20 % of patients [2, 3]. Suboptimal sedation is a commonly seen phenomenon in 75 % patients [4]. Under-sedation occurs in 30 % of patients, resulting in agitated patients demonstrating hypercatabolism and hemodynamic instability with a risk of self-harm and accidental extubation. More commonly, over-sedation from high doses is seen in 40 to 60 % of patients [4]. Over-sedation is linked to slow emergence with a delay in the return of airway reflexes, which prolongs the duration of intubation and mechanical ventilation, thereby increasing the risk of acquiring ventilator-associated pneumonia [4, 5]. Elimination of BDZ, propofol, and opioids rely on good synthetic liver and renal function [5]. Deep sedation is compounded by slow clearance of these agents and systemic drug accumulation among ICU patients who are advancing in age and commonly have liver and renal dysfunction. The daily cost of a ventilated patient ranges $ 3500 to 6000, and thus, the economic impact of additional ventilation days attributable to over-sedation is significant [6].

Sedatives that are currently used are also associated with delirium, which affects up to 80 % of ICU patients [7]. This syndrome is characterized by an acute onset, change or fluctuation in mental status plus inattention, and disorganized thinking or an altered level of consciousness. It is diagnosed at the bedside using the highly reliable tools of the Confusion Assessment Method (CAM-ICU) or Intensive Care Delirium Screening Checklist (ICDSC) [7]. Delirium is associated with a prolonged hospital stay, increased ICU and hospital costs (39 and 31 %, respectively) and contributes to long-term cognitive dysfunction [7–11]. The etiology is multifactorial, but deep sedation (coma) with heavy and prolonged use of BDZ is a well-established risk factor (odds ratio 3.0, 95 % confidence interval 1.3 to 6.8) for its development [8].

Additional concerns surrounding currently used agents include the association of BDZ with the increased long-term risk of developing psychiatric disorders such as anxiety, depression, and post-traumatic stress disorder, which affects more than one-quarter of ICU survivors [12, 13]. The duration of sedation and the use of BDZ are confirmed risk factors for developing all three disorders (mean difference 6.73, 95 % confidence interval 1.42 to 12.06) [12]. Prolonged use of propofol is associated with hypertriglyceridemia, pancreatitis, myoclonus and the rare condition of propofol infusion syndrome, which causes cardiac and renal failure [5, 14]. Over-sedation from these agents, particularly with propofol, also promotes drug-induced hypotension necessitating the use of vasoactive drug support. [5] In 2013, the Society of Critical Care Medicine revised and updated guidelines for the combined management of pain, agitation, delirium (PAD) and recommended the routine assessment of these three interrelated variables, use of lighter sedation regimes, and non-BDZ sedative drugs [5].

The use of volatile anesthetic agents is routinely applied within the operating room to provide general anesthesia. Their use as an ICU sedative appears attractive given these agents are simple to titrate, produce no active metabolites, and are predominantly cleared unchanged by pulmonary exhalation [15]. There are several trials demonstrating these agents promote faster extubation times and better hemodynamic stability with no renal or hepatic toxicity when used for short-term postoperative sedation in comparison to propofol or BDZ sedation [16–18]. Within a RCT of 139 cardiac surgical patients, our group demonstrated that those who received volatile-based anesthesia and postoperative sedation extubated significantly faster by approximately 100 min in comparison to patients who received intravenous propofol [19]. In 2011, Mesnil et al. demonstrated that these agents not only provide faster extubation times but also provide analgesia with a reduction in morphine use and a superior quality of sedation with fewer dose titrations per day when used for up to 96 h [20].

Delivery of volatile agents in the ICU can be simply performed using a small lightweight and portable vaporizer called the Anesthesia Conserving Device (AnaConDa™, Sedana Medical, Sweden). Volatile agents (isoflurane or sevoflurane) are infused into the device, which vaporizes the agent for inhalation. This device is highly efficient and capable of recycling more than 90 % of the expired volatile agent, which facilitates low infusion rates (commonly <5 ml/h) of these agents. This set-up requires scavenging of the volatile gases from the breathing circuit to minimize atmospheric pollution and to ensure workplace safety. Scavenging can be performed within the ICU using charcoal passive absorption or active techniques. [16, 21, 22] Volatile compounds contain fluoride, whose high levels have been historically associated with high-output renal failure caused by an older agent called methoxyflurane, which is no longer used in clinical practice [23]. Studies using the modern volatiles isoflurane or sevoflurane have not shown an association with raised fluoride levels and renal toxicity for limited duration of use [16, 22, 24].

Volatile agents are attractive and theoretically ideal ICU sedatives. However, there is limited experience with the use of these agents for several days. Furthermore, use of these agents and the AnaConDa™ system in critical care units requires staff training and cultural acceptance of this novel modality. Prior to undertaking a large RCT to determine whether these agents provide superior clinical outcomes to our current standard of care, several safety and feasibility issues must be addressed. These issues form the focus of this pilot trial.

## Methods/design

### Study setting

**Fig. 1:**
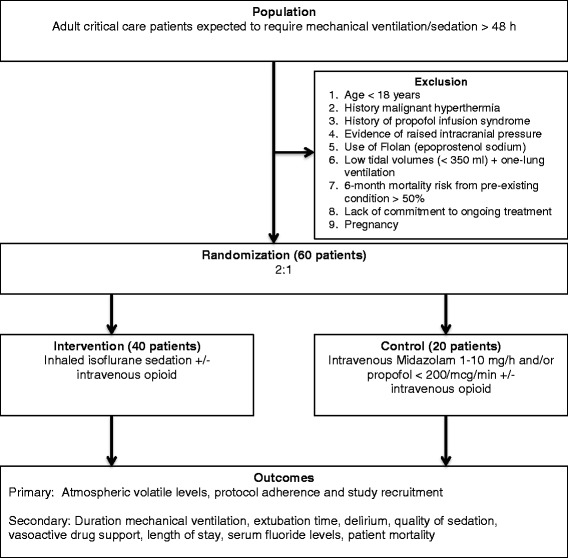
Flow of participants

This is a two-arm prospective multicenter pragmatic pilot RCT comparing the safety and feasibility outcomes of conducting long-term ICU sedation using inhaled isoflurane to standard intravenous propofol and/or midazolam (Fig. 1). This trial was approval by the ethical committee of the Toronto General Hospital, University Health Network (protocol number 13-5845) and University of Ottawa Heart Institute (protocol number 201402500 01H). This clinical trial was originally registered (NCT01983800) on 2 July 2013.

### Eligibility

Patients eligible to be enrolled in this trial are adult ICU patients (>18 years) within 72 h of critical care admission and who are expected to require mechanical ventilation and sedation for longer than 48 h. The following criteria exclude patients from participation in this study: (1) age <18 years; (2) history or family history of malignant hyperthermia; (3) history of propofol infusion syndrome; (4) evidence of raised intracranial pressure; (5) 6-month mortality risk from pre-existing condition >50 %; (6) use of inhaled Flolan (prostacyclin), which is incompatible with the AnaConDa™ device; (7) low tidal volumes (<350 ml) and one-lung ventilation; (8) lack of commitment to ongoing critical care treatment; and (9) pregnancy.

### Recruitment

The study coordinator will assess patients daily for study eligibility within the medical-surgical (MSICU), cardiovascular (CVICU) and coronary (CICU) at TGH and CVICU at Ottawa. Either patient or surrogate decision-maker consent will be obtained prior to study enrollment. Because most patients will be sedated and not have the capacity to provide informed consent, the majority of patients will be consented using surrogate decision-maker consent from the patient’s legal representative.

### Randomization and allocation concealment

This two-arm RCT will randomize 60 patients to the intervention arm, where patients will receive inhaled isoflurane volatile-based sedation, or to the control arm, where the patients will receive standard intravenous midazolam and/or propofol. To further strengthen assessment of the safety of administering volatile agents, patients are allocated 2:1 within the intervention:control groups. Patients will be randomized using a computer sequence generator with permutated blocks (www.randomization.com). To maximize concealment, the size of the block will not be revealed, and randomization will only be performed after patient recruitment. Patients are randomized by the study coordinator, and allocation concealment is maintained by using sequentially numbered, opaque, sealed envelopes.

### Study intervention

Sedation within the two arms of this RCT consists of the following:

- Intervention - Inhaled isoflurane (0 to 5 ml/h)
- Control - Intravenous midazolam (1 to 10 mg/h) and/or propofol (<200 mcg/kg/min)

**Fig. 2:**
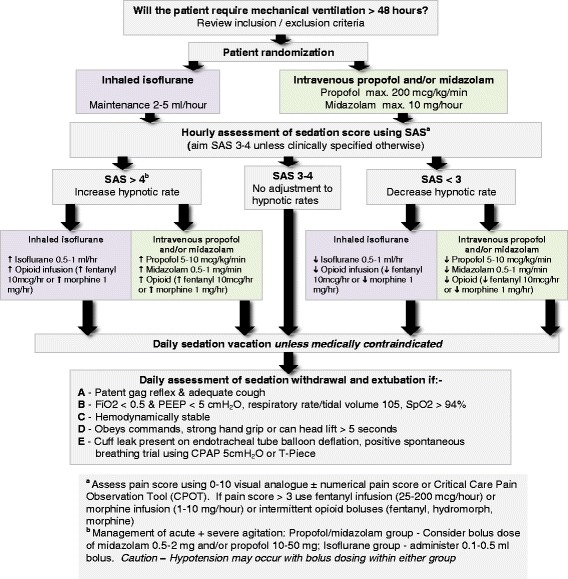
Sedation - analgesia algorithm

Sedation and pain management in both arms will be guided using an explicit bedside sedation-analgesia algorithm (Fig. 2). Sedation in both arms will be titrated every hour to target a Riker Sedation-Agitation Score (SAS) of 3 to 4 (or as clinically indicated) until extubation or tracheostomy. Patients will be reviewed daily for assessment of withdrawing sedation to assist ventilator weaning (resolving the underlying pathology that led to mechanical ventilation; patient capable of making respiratory effort; PaO_2_/FiO_2_ > 150 to 200 mmHg; PEEP 5 to 8 cm H_2_O; satisfactory hemodynamic stability with mean arterial pressure >60 mmHg, which maybe assisted with stable doses of vasoactive drug support; and no evidence of acute myocardial ischemia) and extubated according to the criteria outlined in Fig. 2. Pain scores will be monitored hourly in both groups using the critical care pain observation tool (CPOT) and numerical pain score [5]. Pain will be managed in both arms using intravenous opioids (fentanyl, hydromorphone, or morphine) aiming for pain scores below 3.

### Specialized equipment set-up for volatile sedation

Isoflurane is infused into the AnaConDa™ device, which is placed between the endotracheal tube and the ventilator breathing circuit. The device is changed every 24 h. Isoflurane volatile agent is infused into the device using the Medfusion 3500™ syringe pump driver (Smith Medical, St. Paul, Minnesota, US). A unique and simple syringe driver program for isoflurane has been designed to improve safety of administration. This program includes infusion rate limits in accordance with the sedation protocol, dose range for rapid bolus administration and a keypad automated lock system. Scavenging within the ICU will be performed by standard room air exchanges and active volatile scavenging using our previously described and tested system [21]. This consists of two canisters of Deltasorb™ (Blue-Zone, Concord, Ontario, Canada) assembled in a series from the ventilator expiratory port to the wall outlet suction, which prevents any venting to the atmosphere. Deltasorb™ contains a lattice matrix of silica zeolite, which adsorbs volatile agents. Capnography and end-tidal gas monitoring will be performed at the bedside using the Datex AS/3™ monitor (DRE Inc, Lousiville, Kentucky, US) or Vamos™ monitor (Draeger, Ontario, Canada) monitors. When patients are being prepared for extubation, study sedation drugs will be discontinued, and the AnaConDa™ device will be removed from the breathing circuit, given its high recycling properties, to facilitate a quick drug washout.

### Staff education and training

This trial involves the use of a new mediation and delivery system for ICUs staffed by non-anesthesiologists. Thus, education of medical, nursing and respiratory therapy staff regarding sedation practice and the use of volatile agents is supported by the development of a formal written and lecture-based teaching program. A practical bedside handbook and in-depth teaching manual was specifically designed for this study for critical care teams with limited or no anesthesia experience.^1^ Training sessions include information regarding the roles of the multidisciplinary team, use of the AnaConDa™ device, equipment set-up, trouble-shooting, nebulization of drugs, transport of the critical care patient and safety monitoring. Participants finishing the training session will be completing a confidential questionnaire using a 1 to 5 Likert scale, which will assess the quality of the education. A pre- and post-study questionnaire will be performed to assess staff experience, knowledge, and opinions on using inhalational and intravenous sedation before and after this pilot trial. Nursing and respiratory therapy staff actively involved in the care of enrolled patients will be asked to record their daily satisfaction score regarding both sedation modalities.

### Blinding

Blinding of this study to the healthcare team, patient family and data collectors is not possible given the significant differences in the equipment and bedside monitoring. The data will be blinded to the data analyst.

### Equipment licensing and approvals

The AnaConDa™ device is licensed for use in Canada and Health Canada approval has been granted for the use of isoflurane for critical care sedation (control number 164474).

### Study outcomes

#### Primary outcomes

This pilot trial will primarily assess the sedation-analgesia protocol feasibility and safety by measuring atmospheric volatile concentration levels; see the Project evaluation and data collection for details.

#### Secondary outcomes

The following secondary clinical endpoints will be recorded:

1. Time to extubation (time between discontinuing sedation and tracheal extubation)
2. Duration of mechanical ventilation
3. Quality of sedation assessed by
  - Number hours per day target SAS achieved daily
  - Number of sedation boluses administered per day
  - Opioid requirement
  - Additional sedative and anti-psychotic drugs, for example, morphine BDZ, haloperidol
4. Incidence of delirium
5. Vasopressor/inotropic support
6. Serum fluoride levels (μmol/L)
7. ICU and hospital length of stay
8. ICU and hospital mortality

### Project evaluation and data collection

For patients within the volatile group, daily atmospheric volatile levels (parts per million) will be measured using photometric multigas infrared analyzer (InfraRan™ Specific Vapor Analyzer, Wilkins Enterprise Inc., Massachusetts, USA) to ensure staff safety during volatile use. The InfraRan™ is a bedside gas analyzer, which allows the operator to determine which gas is being measured and calibrated prior to use. Measurement will be performed at four points along the breathing circuit: (1) expiratory limb of the ventilator, (2) post 1st Deltasorb, (3) post 2nd Deltasorb and (4) room levels around the patient’s head [21]. These measurements will be performed by the study coordinator who is trained to use the InfraRan analyzer™. Room levels above 2 ppm indicate high levels of atmospheric pollution, which require immediate equipment assessment for leaks and inadequate room scavenging.

Adherence and violations of the sedation protocol will be assessed by inappropriate equipment set-up and failure to titrate sedation to the target SAS score. This will be assessed by daily equipment checks by a member of the study team. The bedside nurse will record hourly actual and target SAS scores. Protocol violations include no attempt in change in sedative-analgesic regime by the bedside nurse when the actual sedation score fails to meet the target sedation score for more than 2 h. In addition, we will seek feedback and document satisfaction scores from the bedside nurse and respiratory therapist regarding the protocol using a 1 to 5 Likert scale. A screening log of eligible non-randomized patients will also be kept. This will be reviewed to explore reasons for non-enrollment, for example, the study exclusion criteria or refusal by patient’s legal representative or attending physician.

Serum fluoride levels will be measured at 24 h after commencing sedation, followed by further samples every 48 h during sedation with two additional levels measured after discontinuing sedation to assess fluoride washout. Serum fluoride levels will be measured in both treatment groups in order to understand the difference in fluoride levels from volatile sedation; many patients within our two sites have undergone recent surgery where anesthesia is often maintained using volatile agents. The study coordinator will collect 3 to 5 ml of blood in an EDTA tube, which is centrifuged at 2000 g at −30 °C. The plasma is collected and immediately frozen at −70 °C and subsequently fluoride levels are analyzed at a specialized laboratory. Serum fluoride levels will be correlated with the patient’s renal function as assessed using serum creatinine and glomerular filtration rate to look for any associated nephrotoxicity.

Patient demographics, APACHE (Acute Physiology and Chronic Health Evaluation) score, hemodynamic variables and vasoactive drug support, ventilation mechanics, laboratory investigations, clinical ICU complications, length of stay, and mortality will be recorded by daily patient assessment and review of paper and electronic health records. Delirium will be assessed twice daily using the Confusion Assessment Method (CAM-ICU) or Intensive Care Delirium Screening Checklist (ICDSC) [5].

### Sample size and time line

This pilot study will recruit 60 patients over 2 years. Because this pilot trial is designed to mainly assess the feasibility of our protocol and the safety of running volatile agents for ICU sedation, we believe 60 patients will be adequate and in keeping with other pilot studies [25]. This will adequately assess the initial feasibility and safety of using volatile agents for long-term sedation. A total of 66 patients will be recruited to accommodate a 10 % attrition rate. We calculated the sample size for a larger trial based on logarithmic transformation of our preliminary analysis of our short-term sedation results, which demonstrated median extubation times of 178 and 285 min within the volatile and propofol groups, respectively [19]. To demonstrate a moderate Cohen’s effect size with a two-sided α of 0.05 and 90 % power, 170 patients will be required. With an attrition rate of 10 %, the final sample size for randomization is 188 (94 patients per arm).

### Statistical analysis

Continuous parametric variables such as atmospheric volatile levels, serum fluoride levels will be recorded as mean (standard deviation) and analyzed using a two-sample independent *t*-test. All nonparametric data such as satisfaction scores will be recorded as median (interquartile range) and analyzed using the Mann Whitney *U* test. Binary data such as gender, comorbidities will be recorded as proportions and analyzed using the chi-squared or Fishers exact test (if samples <5). Data will be analyzed by an independent statistician using SAS version 9.4 software (Cary, NC, USA). Data analysis will be based on the intention-to-treat principle.

## Discussion

Volatile anesthetics have many pharmacological properties, making it the ideal sedative agent for prolonged duration of use in the ICU. Data from this trial will help us understand the feasibility, acceptance and technical issues associated with conducting volatile sedation in ICUs with little or no experience of this technique. Furthermore, this study will answer important questions regarding fluoride levels during prolonged volatile-based sedation and assess the efficacy of our scavenging system.

### Trial status

This trial is actively enrolling patients. Currently, we have recruited 50 % patients and aim to complete this pilot trial by June 2016.

## Abbreviations

AnaConDa™: Anesthesia Conserving Device
BDZ: benzodiazepines
CAM-ICU: Confusion Assessment Method
ICDSC: Intensive Care Delirium Screening Checklist
ICU: intensive care unit
RCT: randomized controlled trial
SAS: sedation agitation score

## Footnotes

1 VALTS Study Manual. K Wong, A Jerath July 2013
